# Sustainable Methods for Decontamination of Microcystin in Water Using Cold Plasma and UV with Reusable TiO_2_ Nanoparticle Coating

**DOI:** 10.3390/ijerph14050480

**Published:** 2017-05-05

**Authors:** Xuewen Jiang, Seungjun Lee, Chulkyoon Mok, Jiyoung Lee

**Affiliations:** 1 Department of Food Science and Technology, The Ohio State University, Columbus, OH 43210, USA; jiang.1188@osu.edu; 2 Environmental Science Graduate Program, The Ohio State University, Columbus, OH 43210, USA; lee.5178@osu.edu; 3 Department of Food Science and Biotechnology, Gachon University, Seongnam 13557, Korea; mokck@gachon.ac.kr

**Keywords:** *Microcystis aeruginosa*, cyanotoxin, cold plasma, UV, titanium dioxide, emergency preparedness

## Abstract

Microcystins (MCs) are a family of cyanotoxins and pose detrimental effects on human, animal, and ecological health. Conventional water treatment processes have limited success in removing MCs without producing harmful byproducts. Therefore, there is an urgent need for cost-effective and environmentally-friendly methods for treating MCs. The objective of this study was to develop sustainable and non-chemical-based methods for controlling MCs, such as using cold plasma and ultra violet (UV) light with titanium dioxide (TiO_2_) coating, which can be applied for diverse scale and settings. MCs, extracted from *Microcystis aeruginosa*, were treated with cold plasma or UV at irradiance of 1470 μW/cm^2^ (high) or 180 μW/cm^2^ (low). To assess synergistic effects, the outside of the UV treatment chamber was coated with nanoparticles (TiO_2_) prior to irradiation, which can be reused for a long time. The degradation efficiency of UV was enhanced by the reusable TiO_2_ coating at lower irradiance (70.41% [UV] vs. 79.61% [UV+TiO_2_], 120 min), but no significant difference was observed at higher irradiance. Cold plasma removed MCs rapidly under experimental conditions (92%, 120 min), indicating that it is a promising candidate for controlling MCs in water without generating harmful disinfection byproducts. It can be also easily and practically used in household settings during emergency situations.

## 1. Introduction

Microcystins (MCs) refer to a family of cyanotoxins produced by cyanobacteria, such as *Microcystins* spp., *Anabaena* spp., *Nodularia* spp., and several other cyanobacterial species [1]. They are commonly found worldwide in surface waters [2,3]. MCs can cause headache, sore throat, vomiting, nausea, stomachache, diarrhea, and pneumonia in acute exposure [4]. Chronic exposure can lead to liver, kidney, and reproductive system damage and promote cancerous tumors [5,6]. Multiple pathways are involved in MC exposure, such as direct ingestion, inhalation of bioaerosol, or dermal contact of contaminated water [3,7]. According to the World Health Organization (2011) MCs are safe to consume in drinking water at levels of 1 part per billion (ppb) and to exposure in recreational water at 10 ppb [8]. United States Environmental Protection Agency (USEPA) listed MCs as one of the highest priority for health risks in drinking water [9]. New USEPA guidelines for MC-LR (2015) suggest a maximum acceptable concentration in drinking water of 0.3 ppb for pre-school-age-children and 1.6 ppb for school-age-children and adults [10], and 4 ppb for recreational water [11]. However, traditional water treatment steps—including coagulation, sedimentation, and disinfection—have demonstrated limited effect on removing MCs during heavy bloom periods [12]. Furthermore, MCs are resistant to direct UV exposure, ozone, or chlorine oxidation (common steps for disinfection in drinking water treatment) and the toxins may cause health risk for water users [13,14,15,16]. For example, MCs were detected (~2.5 ppb) in the finished drinking water in Toledo, Ohio in August 2014 [10].

Plenty of research has been done on MC degradation using physical, chemical, and biological methods, such as UV irradiation at different wavelength [14,17]; chemical oxidants [16,18], and microcystin-degrading bacteria [19]. However, most previous research focused on a single variant or artificial mixture of MCs, which could have different degradation dynamics and toxicity from natural MC mixture [20]. Therefore, there is a critical need for developing more environmentally-friendly and effective methods for controlling MC contamination to protect both public health and water/food safety, especially because harmful algal blooms are expected to be more frequent and intensive globally [21,22].

In this study, we introduced two novel techniques, cold plasma and nanoparticle application with UV, to examine the effects of both methods for removing MCs in water. Plasma is ionized gas at normal temperature with non-selective short half-life of active species, such as •OH, which can degrade pollutants rapidly without generating toxic residuals [23,24,25,26]. It has been known to be effective to remove chemical contaminants in aqueous phase (e.g., wastewater treatment) [27,28,29], and been used to ensure food safety, especially in fresh produce, by inactivating the microorganisms and removing pesticide residuals [30,31,32]. Barillas et al. (2015) [33] described the capital cost of plasma was 20% less than a traditional wastewater plant. It has been also reported that cold plasma can lower the energy consumption in food industries. However, this technology has not been widely applied for MC degradation. This method only requires water itself during MC treatment, therefore it can be perceived as clean and harmless to both treated objectives and operators, which makes it as a promising alternative to current water treatment methods, possibly for fresh produce application as well.

For nanoparticle application, TiO_2_ has been widely used in wastewater treatment and disinfection. UV illumination induces hydroxyl radicals produced from the active electron and positive hole of TiO_2_, which reacts with pollutants in water [34,35]. It was reported that TiO_2_ showed a great photocatalytic activity to enhance the degradation rate of MC-LR with UV [20,36]. The UV/TiO_2_ wastewater treatment is effective and cost efficient, but increases by further separation of TiO_2_ in the effluent [37]. In this study, the effectiveness of UV with outer coated TiO_2_ has been evaluated to improve the efficiency of entire treatment, as well as lower the cost of separation. TiO_2_ coating is anticipated to be a time- and cost-effective, safe, and sustainable method of MC treatment when combined with UV irradiation.

The objective of this research was to develop and evaluate sustainable and effective methods for MC treatment by cold plasma and UV with TiO_2_. This research describes, for the first time, the effectiveness of cold plasma and UV combined with TiO_2_ coating for total MC (mixture of MC congeners extracted from *M. aeruginosa* that was isolated from Lake Erie) degradation.

## 2. Materials and Methods 

### 2.1. MC Preparation 

MCs were extracted from *Microcystis aeruginosa* (identified by targeting PC-IGS and *mcyA* genes [38]) that was isolated from Lake Erie. *M. aeruginosa* was cultured in CT medium [39] using sonication (three cycles of 5-min sonication followed by 10-min rest) with a Sonic Dismembrator (50 watts, Fisher Scientific Model F50 with Probe, Waltham, MA, USA). Then, three cycles of freeze-thaw lysis were applied, and the extracted solutions was filtered with sterile 0.45 μm pore-size membrane (MF-Millipore Membrane Filter, mixed cellulose esters, Billerica, MA, USA) to remove bacterial cells [40].

### 2.2. MC Treatments and Measurement

**Figure 1:**
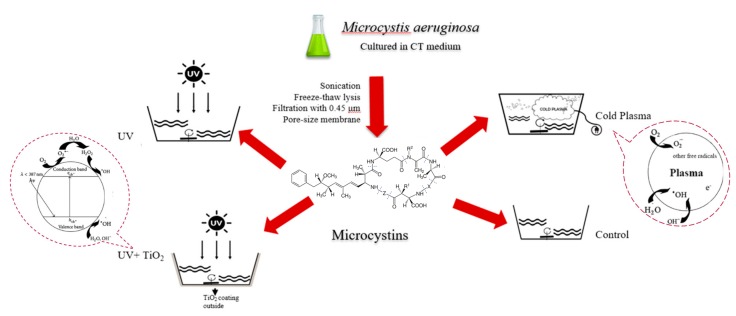
Schematic diagram of MC treatment in this study.

MCs solution was diluted with deionized (DI) water to final concentration of 10 ppb at pH 7.4, EC −17 mV (Mettler-Toledo AG, Analytical. CH-8603, Schwerzenbach, Switzerland). A TiO_2_ nanopowder solution was made in Tween 20 (Boston BioProducts, Ashland, MA, USA): isopropanol (Fisher Scientific, Waltham, MA, USA): acetic acid (Fisher Scientific, USA): TiO_2_ (particle size: 21 nm, Aldrich Chemistry, St. Louis, MO, USA) = 1:45:6:1 by weight) [41], applied ~30 mL of the TiO_2_ nanopowder solution evenly to the outside of a glass container (dimension: 22.5 cm × 33 cm × 5.4 cm [width × length × height], capacity: 2.8 L, materials: tempered soda-lime glass, PYREX_®_), and dried at 25 °C (about two hours) to test the synergistic effect with UV. The UV irradiances were adjusted by the distance between the lamp and targets, and measured with UVC digital light meter (General Tools UV512C, Secaucus, NY, USA) at the central point of the treated water surface. One liter of MC solution was treated with different conditions of application with constant stirring: (1) UV irradiation system (UV-C 254 nm lamp, G15T8, 15 W, Philips, Hamburg, Germany) at 1470 μW/cm^2^ (high irradiance) for 90 min (pre-test showed that MC concentration was below the detection limit after 90 min treatment, data not shown); (2) UV at 180 μW/cm^2^ (low irradiance) for 120 min; (3) UV with TiO_2_ at 1470 μW/cm^2^ (high irradiance) for 90 min (pre-test showed that MC concentration was below the detection limit after 90 min treatment, data not shown); (4) UV with TiO_2_ at 180 μW/cm^2^ (low irradiance) for 120 min; (5) cold plasma (Cleaz one-touch water sterilizer, CSOL-200SL, 220 V, 60 Hz, 80 W, Haan Co., Seoul, Korea) for 120 min; (6) without treatment in dark room for 120 min (control group) (Figure 1). Samples were taken at specific time interval. Total MC concentrations were measured with Microcystins-ADDA enzyme-linked immunosorbent assay (ELISA) kits (Abraxis, Warminster, PA, USA) in duplicate as Ohio EPA described [42]. Under the same conditions, three sets of each type of treatment were performed.

### 2.3. Statistical Analysis

To evaluate toxin degradation, the data were analyzed by one-way analysis of variance (ANOVA) using SPSS Statistics 22 statistical software (IBM, Armonk, NY, USA). For the toxin degradation kinetics, each treatment data was fitted in first- and second-order reaction models.

### 2.4. Energy per Order (E_EO_) of Each Treatment

Energy per order (E_EO_) of each treatment was calculated as the equation below [43].

$$E_{EO}=\frac{Pt\times 1000}{Vlg\left(\frac{C_{i}}{C_{f}}\right)}=\frac{38.4\times P}{V\;k}$$

where *P* is the power of equipment (kW), *V* is the volume of water (L) and *k* is the rate constant calculated in first-order model.

## 3. Results

### 3.1. Synergistic Effects of TiO_2_ with UV on MC Degradation

**Figure 2:**
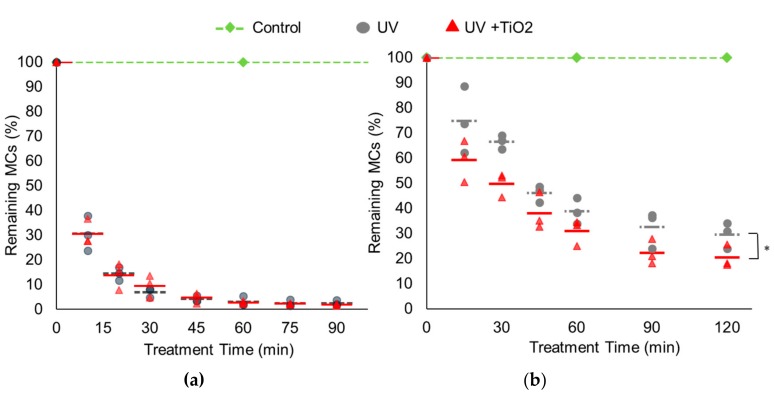
The MC degradation rate in water treated with UV and UV/TiO_2_. Microcystins (final concentration of 10 ppb) were treated with UV (black circle) and UV with TiO_2_ coating outside (red triangle) under high irradiance (1470 μW/cm^2^) for 90 min and low irradiance (180 μW/cm^2^) for 120 min with constant stirring. The control was without UV irradiation in a dark room for 120 min (green diamond). Results represent three independent sets of duplicate measurements. The average of three sets was expressed with line. (**a**) high irradiance (1470 μW/cm^2^); (**b**) low irradiance (180 μW/cm^2^) treatment.

With high irradiance (1470 μW/cm^2^) of UV illumination, 69.5% (10 min), 93.1% (30 min), and 97.6% (90 min) of microcystins in the solution were degraded as the exposure time increased. With the UV and TiO_2_, MCs were removed by 69.4% (10 min), 90.5% (30 min), and 98.1% (90 min), respectively. No significant enhancement by TiO_2_ was observed under this high UV irradiance condition (*p* > 0.05) (Figure 2a).

With low irradiance (180 μW/cm^2^) UV illumination, 33.5% (30 min), 61.3% (60 min), 67.5% (90 min), and 70.4% (120 min) of MCs were degraded as the exposure time increased, but the degrees of MC degradation were lower at each exposure time when compared to the high irradiance (Figure 2b). However, TiO_2_ coating on the outside of the UV treatment container showed a significant improvement in removing MCs at this UV irradiance (*p* < 0.05): 50.2, 69.1, 77.7, and 79.6% at each exposure time, respectively. These results show that UV irradiance is one of the major factors affecting the MCs degradation effectiveness and higher irradiance could accelerate the removal of MCs. However, at lower irradiance, the addition of TiO_2_ significantly enhanced the MC remediation.

### 3.2. Effectiveness of Cold Plasma on MC Degradation

**Figure 3:**
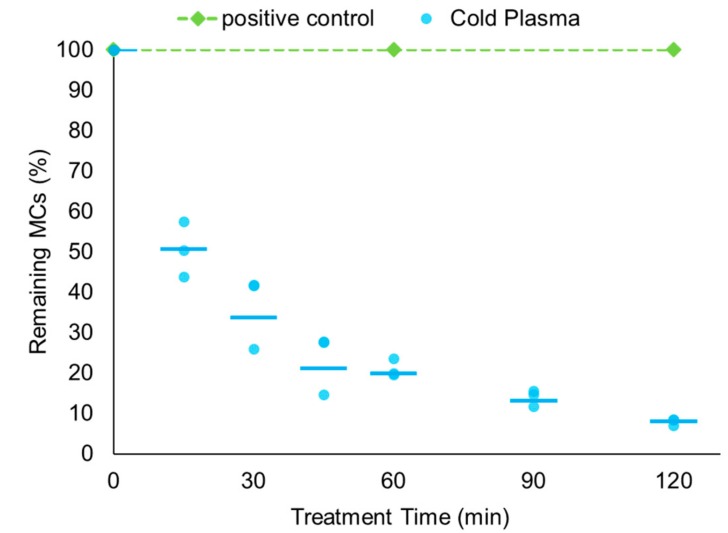
The MC degradation rate in water treated with cold plasma. Microcystins (final concentration of 10 ppb) were treated with cold plasma (blue circle) for 120 min or put in a dark room (as a control) (green diamond) with constant stirring. Results represent three independent sets of duplicate measurements. The average of three sets was expressed as line.

MCs were removed by 66.2% (30 min), 80.2% (60 min), 86.8% (90 min), and 92.0% (120 min) using cold plasma (Figure 3). The MC degradation rate was faster than low irradiance UV illumination (with or without TiO_2_) (*p* < 0.05). This result demonstrates that cold plasma can be a promising candidate for future treatment of MCs. The raw data are shown in Appendix A Table A1.

### 3.3. Kinetic Analysis

To better understand the kinetics of MC degradation with different types of treatments, two different models, first-order-reaction and second-order-reaction, were used, where *k* is the rate constants for the first-order and the second-order reaction kinetics; R^2^ is the corresponding correlation coefficient; t_½_ represents the half-life of microcystins under certain experimental conditions (Table 1 and Table 2).

Both the first-order and second-order models could approximately describe the kinetics of all the treatments (Table 1). It was apparent that the *k* of the high irradiance group of UV treatment was much higher (about four times higher in first-order) than that of the low irradiance group of UV treatment, which indicated that the degradation of MCs by UV irradiation was dose-dependent. After comparing UV with UV/TiO_2_ at low irradiance, the rate constant of the latter was found to be higher and half-lives were shorter, indicating a more rapid degradation with TiO_2_ coating.

For cold plasma, the obtained R^2^ was almost the same for both models (Table 2). The rate constant of cold plasma was 0.0192 min^−1^ in first-order model and 0.0145 (ppb × min) ^−1^ in second-order model, demonstrating a high effectiveness for removing MCs. Compared to low-irradiance UV treatment, cold plasma significantly enhanced the MC degradation rate in both models (*p* < 0.01).

### 3.4. Energy per Order (E_EO_)

Energy cost of each treatment (in first-reaction model) is shown in Table 3. Energy per order (E_EO_) is electrical energy (in kilowatt-hours, kWh) required to reduce the concentration of a pollutant by one order of magnitude (90%) in 1 L of contaminated water [43]. The high irradiance of UV-associated treatments cost the least energy, then followed by cold plasma, UV with TiO_2_ at low irradiance, and UV alone at low irradiance. Overall, as a non-chemical based treatment, the TiO_2_ coating with UV costs less energy than previous methods [45].

## 4. Discussion

Previous research on MC degradation focused on specific variants of MCs, such as MC-LR or RR [46,47,48]. Qiao et al. (2005) demonstrated that H_2_O_2_ enhanced the degradation of MC-RR under UV illumination [47]. In real world situations, a mixture of MC congeners (>100 types) were found to be released into surface water [49], which might suggest different resistance and kinetics to those current treatments. He et al. (2015) [14] investigated the relative susceptibility of various amino acids of MCs to UV treatment (with hydroxyl radical) and they found that MC-YR was the most susceptible and MC-LA was more resistant than MC-LR and -RR. In this study, natural extracts of MCs released from *M. aeruginosa* were used to mimic the toxin residuals in natural blooms, which contains multiple variants of microcystins (detected by LC/MS, data not shown). Ohio EPA (2015) recommended ELISA as a rapid detection method recommended to quantify total microcystins regardless of the congeners, and similar level of MC concentration measured by ELISA, LC-MS/MS, and LC-UV was obtained [42,50]. Therefore, due to its ability to measure multiple MC variants, ELISA was one of the best options to collectively measure the MCs.

UV, as a traditional disinfection method in water treatment plants, has been evaluated for MC degradation [51]. As the results herein show, the irradiance of UV highly affects its effectiveness. At high irradiance, UV alone could rapidly degrade MCs in a short time, whereas a low irradiance UV less effectively removed MCs. However, the general requirement of UV irradiance for water treatment is two order of magnitude lower than the experimental condition of high irradiance. Previous studies added TiO_2_ directly into the water that is treated as a catalyst. TiO_2_ in the water may cause another contamination problem since there might be unpredictable hazards when exposed to the excessive TiO_2_ and using the TiO_2_ increases the treatment cost. In this study, the synergistic effect of TiO_2_ under UV irritation was tested by coating the outer surface of the container, which improved public perception by preventing the release of the TiO_2_ into the treated water. In addition, this type of application maximizes the reusability of TiO_2_, thereby it can reduce the cost of each treatment and lower the risk of environmental contamination of these nanoparticles. Meanwhile, the coating method saved the cost for removing the nanoparticles from the treated water compared to other TiO_2_ treatment method (nanoparticles were added in water). The saved cost was not reflected in the E_EO_ calculation. As shown in the results, lower UV dose was still effective in removing MCs due to the synergistic effect of TiO_2_ (additional 17% (30 min) and 9% MC (120 min) removal). Therefore, TiO_2_ coating could shorten UV irradiation time to achieve a desired MC degradation efficiency at a relatively low UV irradiance. In addition, compared to Sampaio et al. (2015) [45] (high irradiance of UV (47,100 μW/cm^2^) with TiO_2_ into water directly), our remediation treatment (UV at 180 μW/cm^2^ with surface coated TiO_2_) was still effective (constant of first-order reaction) in removing MC degradation in water (Table 1). Therefore, the coating method can be considered as an alternative to direct addition when removing cyanotoxins in a sustainable and environmental friendly way in various water treatment settings.

Cold plasma is a safe and rapid treatment that is applicable for various situations with different scales and materials [44]. Compared to chemical-based methods, such as chlorination, Zhang et al. (2012) [44] demonstrated that the intermediates and end-products produced during MC degradation by plasma treatment were non-toxic. Previous studies [44,48] demonstrated that plasma was effective for removal of MC-LR by attacking the conjugated carbon double bonds of the Adda structure. Zhang et al. (2012) [44] showed that glow discharge plasma can degrade MC-LR with a rate constant of 38.81 ppb^−1^ min^−1^ in second-order model (Table 2). This study is the first to confirm the effectiveness in MC degradation while using the natural mixture of MCs derived from the toxic *M. aeruginosa* that was isolated from the harmful bloom in Lake Erie.

When compared with the UV+TiO_2_ (low UV irradiance), the cold plasma treatment showed additional 16% (30 min) and 12% (120 min) of MCs removal at the same exposure times under the study conditions. Therefore, cold plasma treatment can shorten the MC treatment time further, which will be quite useful and impactful, especially during a high bloom period and under an emergency when toxins in the water need to be treated in a timely manner. The E_EO_ of cold plasma was lower than UV and UV/TiO_2_ at low irradiance. The treatment time can be further shortened if the levels of MC are relatively low, indicating that it could serve as a practical backup for family treatment. For example, when municipal water treatment cannot meet the USEPA guideline in drinking water (e.g., Toledo water crisis in 2014), cold plasma can be immediately used to treat the MCs in tap water in affected families. In water treatment plants, large-scale application might improve effectiveness further to achieve the same degradation effects in a shorter period. Previous studies demonstrated that powerful disinfection using cold plasma in fresh produce can be achieved without generating extra heat and destruction of food quality [23,52,53,54]. Moreover, the cold plasma did not require any reagents beyond air and water, therefore it is a green technology without generation of harmful waste and unwanted byproducts. However, few studies have been conducted on investigating the feasibility of cold plasma application in MC removal in food, which is another critical exposure route to cyanotoxins [55]. Combining the results from this study, it is promising to test the effectiveness of cold plasma for degrading MCs accumulated in fresh produce, fish, and other food-related matrices. Therefore, more efforts should be made in MC decontamination in food in the future.

## 5. Conclusions

This study reports the effectiveness of total MC degradation using sustainable cold plasma and TiO_2_ coating. The UV irradiation combined with TiO_2_-coating can enhance the effectiveness of MC degradation. Our developed technique prevents the direct contact between TiO_2_ and water to avoid unwanted contamination. In addition, the cold plasma degraded the toxin effectively without generating harmful byproducts. The cold plasma can be a promising application for both household and industry settings, using different scales of equipment. In future studies, other analytical tools other than ELISA and toxicity tests are recommended to identify the potential changes in the composition of microcystins and intermediates after each type of toxin treatment. It can also be practically used in household settings during emergency situations.

## Figures and Tables

**Table 1:** Degradation rate constants (*k*) and half-lives (t_½_) in the first- or second-order reaction kinetics in microcystin removal by UV and UV with TiO_2_.

Treatment	First-order (Ln(C_0_/C) = −*k*t)			Reference
	*k* (min^−1^)	t_½ _(min)	R^2^	
UV (Irradiance: 1470 μW/cm^2^)	0.0394	17.59	0.89	This study
UV with TiO_2_ (Irradiance: 1470 μW/cm^2^)	0.0412	16.82	0.91	This study
UV (Irradiance: 180 μW/cm^2^)	0.0104	66.64	0.92	This study
UV with TiO_2_ (Irradiance: 180 μW/cm^2^)	0.0129	53.73	0.93	This study
UV with TiO_2_ added (~182 ppb MC-LA; 47,100 μW/cm^2^)	0.0277	25.02	0.99	[29]
**Treatment**	**Second-order (1/C − 1/C_0_ = *k*t)**			**Reference**
	***k* (ppb × min)^−1^**	**t_½ _(min)**	**R^2^**	
UV (Irradiance: 1470 μW/cm^2^)	0.0848	1.74	0.92	This study
UV with TiO_2_ (Irradiance: 1470 μW/cm^2^)	0.0913	1.58	0.95	This study
UV (Irradiance: 180 μW/cm^2^)	0.0025	46.34	0.91	This study
UV with TiO_2_ (Irradiance: 180 μW/cm^2^)	0.0028	28.09	0.93	This study

**Table 2:** Degradation rate constants (*k*) and half-lives (t_½_) in the first- or second-order reaction kinetics in microcystin removal by cold plasma.

Treatment	First-order (Ln(C_0_/C) = −*k*t)			Reference
	*k *(min^−1^)	t_½_ (min)	R^2^	
Cold plasma	0.0192	36.10	0.95	This study
**Treatment**	**Second-order (1/C − 1/C_0_ = * k* t)**			**Reference**
	***k* (ppb × min)^−1^**	**t_½_ (min)**	**R^2^**	
Cold plasma	0.0150	10.88	0.93	This study
Glow discharge plasma (4025.5 ppb MC-LR)	38.81	0.018	Not mentioned	[44]

**Table 3:** Energy per order (E_EO_) of each treatment.

Treatment	E_EO_ (kWh/m^3^/order)	Reference
UV (Irradiance: 1470 μW/cm^2^)	27.96	This study
UV with TiO_2_ (Irradiance: 1470 μW/cm^2^)	29.85	This study
UV (Irradiance: 180 μW/cm^2^)	110.73	This study
UV with TiO_2_ (Irradiance: 180 μW/cm^2^)	89.26	This study
UV with TiO_2_ added	415.71	[45]
Cold plasma	59.97	This study

## Appendix A

**Table A1:** Concentrations of MC remained in the water after the treatments.

		Time (min)	0	30	90	120
	Concentrstion (ppb)					
Treatment						
**UV (Irradiance: 1470 μW/cm^2^)**			6.76 ± 1.50	0.48 ± 0.15	0.16 ± 0.03	N/A
**UV with TiO_2_ (Irradiance: 1470 μW/cm^2^)**			6.95 ± 0.92	0.66 ± 0.34	0.13 ± 0.016	
**UV (Irradiance: 180 μW/cm^2^)**			8.63 ± 0.0	5.74 ± 0.24	2.81 ± 0.64	2.55 ± 0.45
**UV with TiO_2_ (Irradiance: 180 μW/cm^2^)**			12.72 ± 0.0	6.33 ± 0.61	2.83 ± 0.63	2.59 ± 0.58
**Cold plasma**			6.13 ± 0.78	2.20 ± 0.45	0.85 ± 0.12	0.50 ± 0.11
